# Supplementary figures and images for: Comparative evaluation of TNM staging systems (eighth vs. ninth edition) for the non-surgical treatment of localized and locally advanced anal squamous cell carcinoma: Prognostic significance of T classification and lymph node status
Source: PLoS One. 2025 Jan 16;20(1):e0317598. doi: 10.1371/journal.pone.0317598 (PMC11737782; doi:10.1371/journal.pone.0317598)

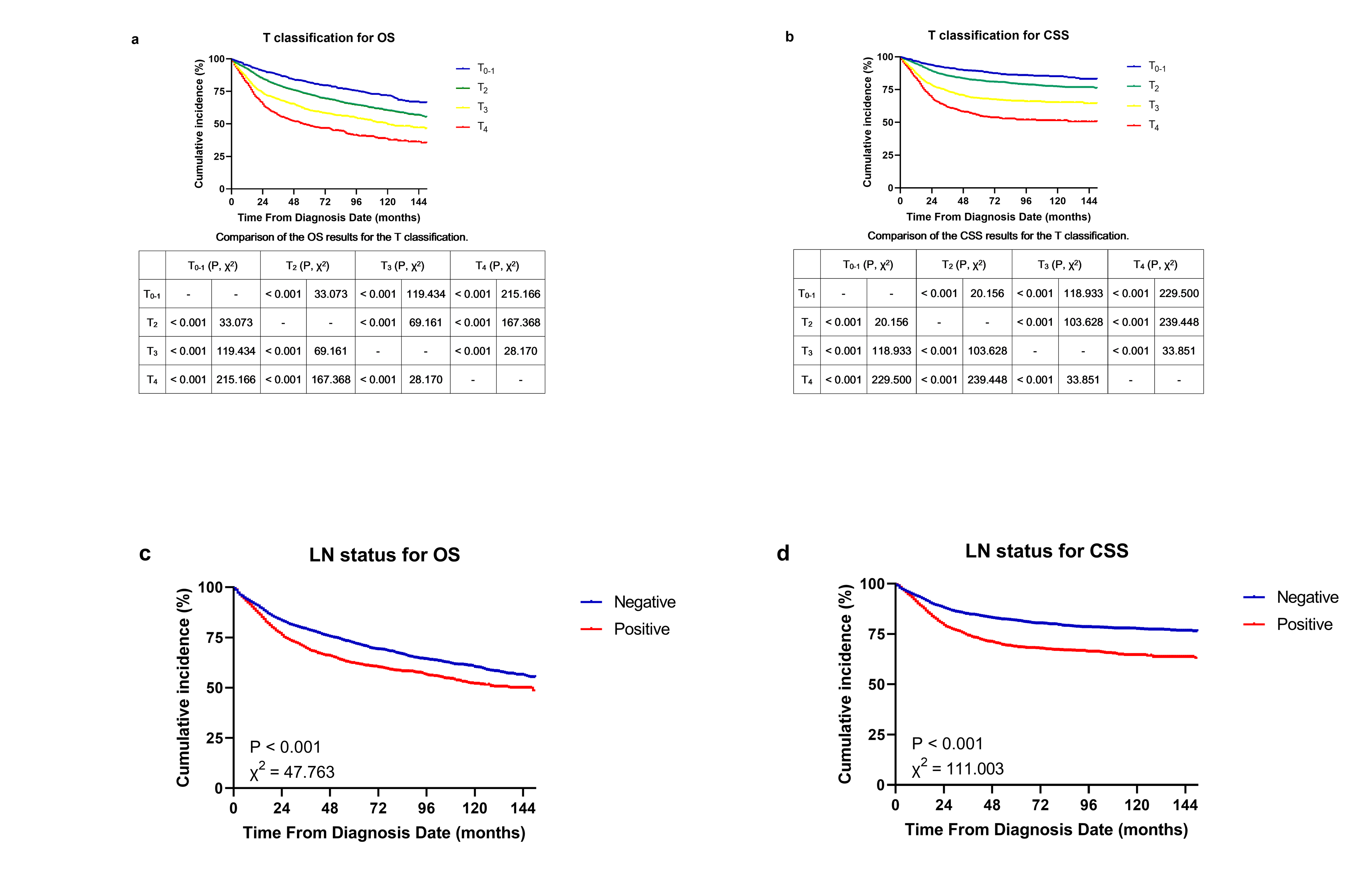

Supplement: S1 Fig — OS and CSS of patients with LLA ASCC who received non-surgical treatment according to the (a, b) T classification and (c, d) LN status. (TIF) [file pone.0317598.s001.tif]

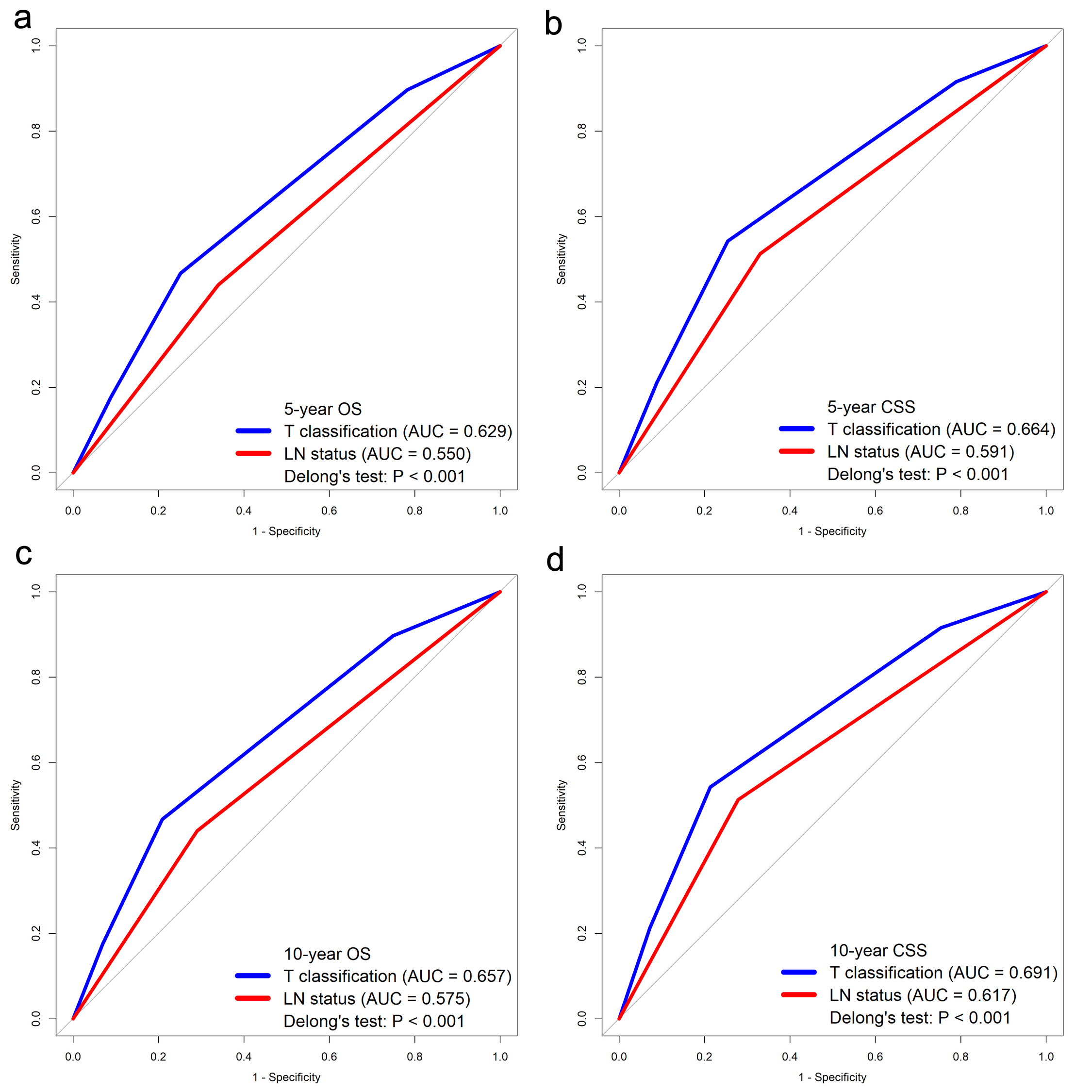

Supplement: S2 Fig — tdROC curves of the T classification and LN status at (a, b) 5-year OS and CSS, and (c, d) 10-year OS and CSS. (TIF) [file pone.0317598.s002.tif]
