# Supplementary material for: Comparative evaluation of TNM staging systems (eighth vs. ninth edition) for the non-surgical treatment of localized and locally advanced anal squamous cell carcinoma: Prognostic significance of T classification and lymph node status
Source: PLoS One. 2025 Jan 16;20(1):e0317598. doi: 10.1371/journal.pone.0317598 (PMC11737782; doi:10.1371/journal.pone.0317598)
Supplement: S1 Table — (DOCX) [file pone.0317598.s003.docx]

S1 Table. Univariate and multivariate Cox regression analysis of OS and CSS with TNM eighth and ninth editions.

|  | Overall survival (OS) | | | | | | | |  | Cause-specific survival (CSS) | | | | | | | |
| --- | --- | --- | --- | --- | --- | --- | --- | --- | --- | --- | --- | --- | --- | --- | --- | --- | --- |
|  | Univariate | |  | Multivariate, TNM8th | |  | Multivariate, TNM9th | |  | Univariate | |  | Multivariate, TNM8th | |  | Multivariate, TNM9th | |
| *Factor* | ***P****-value* | *HR (95% CI)* |  | ***P****-value* | *HR (95% CI)* |  | ***P****-value* | *HR (95% CI)* |  | ***P****-value* | *HR (95% CI)* |  | ***P****-value* | *HR (95% CI)* |  | ***P****-value* | *HR (95% CI)* |
| *Age at diagnosis, ≤ 60 vs. > 60* | < 0.001 | 1.669 (1.533-1.818) |  | < 0.001 | 1.732 (1.588-1.890) |  | < 0.001 | 1.732 (1.588-1.889) |  | 0.003 | 1.172 (1.056-1.300) |  | < 0.001 | 1.241 (1.115-1.380) |  | < 0.001 | 1.237 (1.112-1.376) |
| *Gender, Female vs. Male* | < 0.001 | 1.584 (1.452-1.727) |  | < 0.001 | 1.716 (1.571-1.873) |  | < 0.001 | 1.761 (1.612-1.923) |  | < 0.001 | 1.717 (1.545-1.909) |  | < 0.001 | 1.814 (1.628-2.021) |  | < 0.001 | 1.882 (1.688-2.098) |
| *Race, White vs. Non-white* | < 0.001 | 1.244 (1.105-1.401) |  | 0.004 | 1.195 (1.060-1.347) |  | 0.003 | 1.201 (1.065-1.353) |  | < 0.001 | 1.336 (1.158-1.542) |  | 0.020 | 1.189 (1.028-1.374) |  | 0.013 | 1.202 (1.040-1.389) |
| *TNM8th edition, I* | *reference* |  |  | *reference* |  |  | - |  |  | *reference* |  |  | *reference* |  |  | - |  |
| IIA | < 0.001 | 1.477 (1.255-1.739) |  | < 0.001 | 1.574 (1.337-1.854) |  |  |  |  | < 0.001 | 1.556 (1.232-1.964) |  | < 0.001 | 1.686 (1.335-2.130) |  |  |  |
| IIB | < 0.001 | 2.478 (2.044-3.004) |  | < 0.001 | 2.560 (2.111-3.104) |  |  |  |  | < 0.001 | 3.356 (2.589-4.350) |  | < 0.001 | 3.468 (2.674-4.497) |  |  |  |
| IIIA | < 0.001 | 1.582 (1.321-1.895) |  | < 0.001 | 1.826 (1.524-2.189) |  |  |  |  | < 0.001 | 2.200 (1.720-2.813) |  | < 0.001 | 2.504 (1.956-3.204) |  |  |  |
| IIIB | < 0.001 | 2.979 (2.402-3.694) |  | < 0.001 | 3.098 (2.497-3.844) |  |  |  |  | < 0.001 | 4.217 (3.181-5.590) |  | < 0.001 | 4.397 (3.314-5.834) |  |  |  |
| IIIC | < 0.001 | 2.601 (2.207-3.066) |  | < 0.001 | 2.964 (2.513-3.496) |  |  |  |  | < 0.001 | 3.920 (3.129-4.912) |  | < 0.001 | 4.390 (3.500-5.505) |  |  |  |
| *TNM9th edition, I* | *reference* |  |  | *-* |  |  | *reference* |  |  | *reference* |  |  | - |  |  | *reference* |  |
| IIA | < 0.001 | 1.477 (1.255-1.739) |  |  |  |  | < 0.001 | 1.573 (1.336-1.852) |  | < 0.001 | 1.556 (1.232-1.964) |  |  |  |  | < 0.001 | 1.684 (1.333-2.127) |
| IIB | < 0.001 | 1.582 (1.321-1.895) |  |  |  |  | < 0.001 | 1.828 (1.526-2.191) |  | < 0.001 | 2.201 (1.721-2.814) |  |  |  |  | < 0.001 | 2.507 (1.959-3.209) |
| IIIA | < 0.001 | 2.340 (1.986-2.758) |  |  |  |  | < 0.001 | 2.538 (2.152-2.992) |  | < 0.001 | 3.311 (2.639-4.154) |  |  |  |  | < 0.001 | 3.554 (2.831-4.462) |
| IIIB | < 0.001 | 2.980 (2.403-3.695) |  |  |  |  | < 0.001 | 3.116 (2.511-3.865) |  | < 0.001 | 4.219 (3.182-5.593) |  |  |  |  | < 0.001 | 4.436 (3.344-5.886) |
| IIIC | < 0.001 | 3.490 (2.877-4.234) |  |  |  |  | < 0.001 | 4.215 (3.470-5.120) |  | < 0.001 | 5.636 (4.387-7.242) |  |  |  |  | < 0.001 | 6.685 (5.196-8.601) |
| *RT, No/Unknown vs. Yes* | < 0.001 | 0.312 (0.273-0.357) |  | < 0.001 | 0.555 (0.471-0.655) |  | < 0.001 | 0.573 (0.486-0.675) |  | < 0.001 | 0.253 (0.217-0.295) |  | < 0.001 | 0.431 (0.355-0.524) |  | < 0.001 | 0.449 (0.370-0.545) |
| *CTx, No/Unknown vs. Yes* | < 0.001 | 0.314 (0.281-0.350) |  | < 0.001 | 0.407 (0.356-0.456) |  | < 0.001 | 0.401 (0.351-0.458) |  | < 0.001 | 0.300 (0.263-0.343) |  | < 0.001 | 0.420 (0.355-0.497) |  | < 0.001 | 0.410 (0.347-0.485) |

RT: radiotherapy; CTx: chemotherapy; HR, hazard ratio; CI, confidence interval.
