# Supplementary material for: Comparative evaluation of TNM staging systems (eighth vs. ninth edition) for the non-surgical treatment of localized and locally advanced anal squamous cell carcinoma: Prognostic significance of T classification and lymph node status
Source: PLoS One. 2025 Jan 16;20(1):e0317598. doi: 10.1371/journal.pone.0317598 (PMC11737782; doi:10.1371/journal.pone.0317598)
Supplement: S2 Table — (DOCX) [file pone.0317598.s004.docx]

S2 Table. Univariate and multivariate Cox regression analysis of OS and CSS with the T classification and LN status.

|  | Overall survival (OS) | | | | |  | Cause-specific survival (CSS) | | | | |
| --- | --- | --- | --- | --- | --- | --- | --- | --- | --- | --- | --- |
|  | Univariate | |  | Multivariate | |  | Univariate | |  | Multivariate | |
| *Factor* | ***P****-value* | *HR (95% CI)* |  | ***P****-value* | *HR (95% CI)* |  | ***P****-value* | *HR (95% CI)* |  | ***P****-value* | *HR (95% CI)* |
| *Age at diagnosis, ≤ 60 vs. > 60* | < 0.001 | 1.669 (1.533-1.818) |  | < 0.001 | 1.727 (1.583-1.884) |  | 0.003 | 1.172 (1.056-1.300) |  | < 0.001 | 1.234 (1.110-1.373) |
| *Gender, Female vs. Male* | < 0.001 | 1.584 (1.452-1.727) |  | < 0.001 | 1.758 (1.610-1.920) |  | < 0.001 | 1.717 (1.545-1.909) |  | < 0.001 | 1.875 (1.682-2.090) |
| *Race, White vs. Non-white* | < 0.001 | 1.244 (1.105-1.401) |  | < 0.001 | 1.199 (1.063-1.351) |  | < 0.001 | 1.336 (1.158-1.542) |  | 0.015 | 1.198 (1.036-1.385) |
| *T stage, T0-1* | *reference* |  |  | *reference* |  |  | *reference* |  |  | *reference* |  |
| T2 | < 0.001 | 1.509 (1.306-1.745) |  | < 0.001 | 1.573 (1.360-1.820) |  | < 0.001 | 1.552 (1.274-1.890) |  | < 0.001 | 1.608 (1.319-1.960) |
| T3 | < 0.001 | 2.300 (1.976-2.678) |  | < 0.001 | 2.247 (1.917-2.633) |  | < 0.001 | 2.925 (2.395-3.573) |  | < 0.001 | 2.611 (2.119-3.218) |
| T4 | < 0.001 | 3.215 (2.727-3.789) |  | < 0.001 | 3.330 (2.813-3.943) |  | < 0.001 | 4.455 (3.607-5.502) |  | < 0.001 | 4.301 (3.460-5.345) |
| *LN status, Negative vs. Positive* | < 0.001 | 1.344 (1.235-1.462) |  | < 0.001 | 1.179 (1.076-1.293) |  | < 0.001 | 1.733 (1.562-1.922) |  | < 0.001 | 1.381 (1.233-1.547) |
| *RT, No/Unknown vs. Yes* | < 0.001 | 0.312 (0.273-0.357) |  | < 0.001 | 0.571 (0.484-0.674) |  | < 0.001 | 0.253 (0.217-0.295) |  | < 0.001 | 0.449 (0.370-0.545) |
| *CTx, No/Unknown vs. Yes* | < 0.001 | 0.314 (0.281-0.350) |  | < 0.001 | 0.402 (0.351-0.459) |  | < 0.001 | 0.300 (0.263-0.343) |  | < 0.001 | 0.408 (0.346-0.483) |

RT: radiotherapy; CTx: chemotherapy; HR, hazard ratio; CI, confidence interval.
